# Cellular and humoral immunogenicity against SARS-CoV-2 vaccination or infection is associated with the memory phenotype of T- and B-lymphocytes in adult allogeneic hematopoietic cell transplant recipients

**DOI:** 10.1007/s12185-024-03802-3

**Published:** 2024-06-06

**Authors:** Takaaki Konuma, Megumi Hamatani-Asakura, Etsuko Nagai, Eisuke Adachi, Seiko Kato, Masamichi Isobe, Maki Monna-Oiwa, Satoshi Takahashi, Hiroshi Yotsuyanagi, Yasuhito Nannya

**Affiliations:** 1 Department of Hematology/Oncology, The Institute of Medical Science, The University of Tokyo, 4-6-1, Shirokanedai, Minato-ku, Tokyo, Japan; 2 Department of Laboratory Medicine, The Institute of Medical Science, The University of Tokyo, Tokyo, Japan; 3 Department of Infectious Diseases and Applied Immunology, The Institute of Medical Science, The University of Tokyo, Tokyo, Japan; 4 Division of Clinical Precision Research Platform, The Institute of Medical Science, The University of Tokyo, Tokyo, Japan

**Keywords:** Allogeneic hematopoietic cell transplantation, Coronavirus disease 2019 (COVID-19), Memory B cells, Severe acute respiratory syndrome coronavirus 2 (SARS-CoV-2), Memory T cells

## Abstract

We conducted a cross-sectional study to evaluate cellular and humoral immunogenicity against severe acute respiratory syndrome coronavirus 2 (SARS-CoV-2) vaccination or infection and examine how lymphocyte subpopulations in peripheral blood correlate with cellular and humoral immunogenicity in adult allogeneic hematopoietic cell transplantation (HCT) recipients. The median period from SARS-CoV-2 vaccination or infection to sample collection was 110.5 days (range, 6–345 days). The median SARS-CoV-2 spike-specific antibody level was 1761 binding antibody units (BAU)/ml (range, 0 to > 11,360 BAU/ml). Enzyme-linked immunosorbent spot (ELISpot) assay of T cells stimulated with SARS-CoV-2 spike antigens showed that interferon-gamma (IFN-γ)-, interleukin-2 (IL-2)-, and IFN-γ + IL-2-producing T cells were present in 68.9%, 62.0%, and 56.8% of patients, respectively. The antibody level was significantly correlated with frequency of IL-2-producing T cells (*P* = 0.001) and IFN-γ + IL-2-producing T cells (*P* = 0.006) but not IFN-γ-producing T cells (*P* = 0.970). Absolute counts of CD8+ and CD4+ central memory T cells were higher in both IL-2- and IFN-γ + IL-2-producing cellular responders compared with non-responders. These data suggest that cellular and humoral immunogenicity against SARS-CoV-2 vaccination or infection is associated with the memory phenotype of T cells and B cells in adult allogeneic HCT recipients.

**Supplementary Information:**

The online version contains supplementary material available at 10.1007/s12185-024-03802-3.

## Introduction

Severe acute respiratory syndrome coronavirus 2 (SARS-CoV-2) is the cause of the novel coronavirus disease 2019 (COVID-19), which spread worldwide as a global pandemic [1, 2]. Vaccinations against SARS-CoV-2 were proven to be effective in the reduction of COVID-19 infection and mortality in the general population [3, 4].

Patients with hematological diseases, including those who received allogeneic hematopoietic cell transplantation (HCT), are at higher risk of severity and mortality of COVID-19 than the general population [5–9]. This is partly because the immune response to SARS-CoV-2 vaccination or infection is impaired in these populations [10]. Several studies have shown that a SARS-CoV-2-specific T-cell response as well as antibody levels play an important role in protection after vaccination or infection [11–14]. However, although most early studies evaluated the humoral response against SARS-CoV-2 vaccination or infection mainly by quantitative measurement of antibody concentration in serum [15–32], data on the cellular response are still scarce, particularly in adults receiving allogeneic HCT [33–43]. Furthermore, SARS-CoV-2-specific T-cell memory has been detected with a predominant interleukin-2 (IL-2) memory response compared to interferon-gamma (IFN-γ) [44, 45]. Here, we performed a comprehensive analysis of both cellular and humoral immunity using a dual-color enzyme-linked immunosorbent spot (ELISpot) assay capable of detecting both IL-2 and IFN-γ secreting T cells in response to SARS-CoV-2 antigens in adult patients who had received allogeneic HCT.

The primary objective of this study was to evaluate the correlation between cellular and humoral immune responses against SARS-CoV-2 vaccination or infection and the associated factors in adults receiving allogeneic HCT. The secondary objective was to evaluate the association between peripheral blood lymphocyte subpopulations and SARS-CoV-2 antibody levels and frequencies of SARS-CoV-2-specific T cells in this population.

## Methods

### Patients

This cross-sectional study was conducted at the Institute of Medical Science, University of Tokyo. Fifty-eight patients who had undergone outpatient treatment without hematological disease recurrence for at least 3 months after allogeneic HCT were recruited at our institute between February 2022 and September 2022. Samples from 7 healthy subjects without hematological disease were used as control samples. Written informed consent was acquired from all patients. The Institutional Review Board of the Institute of Medical Science, University of Tokyo approved this study (2021-64-1216). This study was conducted in accordance with the Declaration of Helsinki.

### Antibody detection against SARS-CoV-2

Serum samples were tested for the presence of quantitative spike (S) IgG antibodies against SARS-CoV-2, including neutralizing antibodies, which bind to the receptor binding domain of the S1 subunit of the S protein, by chemiluminescent immunoassay using an Alinity SARS-CoV-2 IgG II Quant assay (Abbott, Abbott Park, IL), according to the manufacturer’s instructions [46]. The results are reported in binding antibody units/ml (BAU/ml) calculated by multiplying the Abbott AU/ml by a conversion factor of 0.142, as described in the previous report [47]. Humoral responses were considered positive for antibody levels ≥ 250 BAU/ml, which was associated with > 90% efficacy of mRNA-1273, as previously described [48].

### Peripheral blood sample collection

Peripheral blood mononuclear cells (PBMCs) were obtained by Lymphoprep (Axis-Shield PoC, Oslo) density-gradient centrifugation and stored in Bambanker cryopreservation medium (Nippon Genetic Co. Ltd., Tokyo) until analysis.

### ELISpot assay for IFN-γ and IL-2

The ELISpot assays were performed using a human IFN-γ /IL-2 double-color enzymatic kit (hIFNGIL2-1M, Cellular Technology Ltd [CTL]., Shaker Heights, OH) according to the manufacturer’s protocol. The polyvinylidene difluoride membranes were pre-wet with 70% ethanol, and the plates were coated with human IFN-γ and IL-2 coating antibodies overnight. The next day, 96-well plates were washed once with phosphate-buffered saline, and PBMCs were plated at 200,000 cells per well with SARS-CoV-2 S1 scanning pool, which contains 166 peptides from the 15-mer peptides overlapping with 11 amino acids, covering the S1 subunit of the spike protein of SARS-CoV-2 (amino acids 13–685) (final concentration of 2 μg/ml, 3629-1, Mabtech AB, Sweden) in 200 μl of CTL-Test Medium. Anti-human CD28 antibody (final concentration of 0.1 μg/ml, 302934, BioLegend, San Diego, CA) was added for stimulation for T cells. Negative control wells lacked peptides, and positive control wells contained phorbol myristate acetate (PMA), ionomycin (IM), brefeldin A, and monensin in the Cell Stimulation Cocktail (Tonbo Biosciences, San Diego, CA). After 16–18 h of incubation in a humidified incubator at 37 °C with 5% CO_2_, the PBMCs were removed, and the detection antibodies and development reagents were added. Air‐dried plates were scanned and analyzed using an ImmunoSpot® S6 Universal Analyzer (CTL). Spot forming units (SFU) were automatically calculated by the ImmunoSpot® Software 7.0.34.0 for antigen stimulation conditions. All tests were performed in duplicate or triplicate, and the mean values of duplicated or triplicated cell cultures were considered. Cellular responses were considered positive for stimulated spot numbers > threefold higher than negative controls combined with an increment value of > 3 SFU per well (200,000 cells) as previously described [49].

### Lymphocyte subsets analysis

We separately analyzed T-cell and B-cell lymphocyte subsets. For T-cell subset analysis, PBMCs were stained with Alexa Fluor (AF) 700-conjugated anti-human CD3 (clone UCHT-1, BioLegend), Brilliant Violet (BV) 510-conjugated anti-human CD4 (clone RPA-T4, BioLegend), fluorescein isothiocyanate (FITC)-conjugated anti-human CD8 (clone RPA-T8, Tonbo Biosciences), allophycocyanin (APC)-Cy7-conjugated anti-human CD45RA (clone HI100, BioLegend), and BV421-conjugated anti-human CCR7 (clone G043H7, BioLegend) antibodies. For the B-cell subset analysis, PBMCs were also stained with AF700-conjugated anti-human CD3 (clone UCHT-1, BioLegend), APC-Cy7-conjugated anti-human CD19 (clone HIB19, BioLegend), FITC-conjugated anti-human CD27 (clone O323, T BioLegend), BV421-conjugated anti-human IgD (clone IA6-2, BioLegend), phycoerythrin (PE)-conjugated anti-human CD24 (clone ML5, BioLegend), and PE-Cy7-conjugated anti-human CD38 (clone HIT2, BioLegend) antibodies. The gating strategy for lymphocyte subpopulations, as previously described [50], is shown in Supplementary Table 1. The analysis was performed on a BD FACSCelesta (BD Bioscience, San Jose, CA).

### Statistical analysis

All statistical analyses were performed using GraphPad Prism 9 or 10 for Mac OS X (GraphPad Software Inc, San Diego, CA). Continuous variables were compared using Mann–Whitney *U* test or the Kruskal–Wallis test. The Spearman rank correlation coefficient was calculated to assess the correlation between continuous variables.

In the univariate analysis, SARS-CoV-2-specific antibody levels and T-cell SFU were stratified by age at sample collection (< 55 vs. ≥ 55 years), gender (male vs. female), disease type (myeloid malignancies vs. others), graft source (bone marrow vs. cord blood [CB]), chronic graft-versus-host disease (GVHD) (none, non-active vs. active), immunosuppressive treatment (IST) (with vs. without), interval from HCT to SARS-CoV-2 vaccination or infection (< 2 vs. ≥ 2 years), SARS-CoV-2 status (2nd, 3rd, 4th vaccination vs. infection), vaccine type (mRNA-1273 vs. BNT162b2), and interval from SARS-CoV-2 vaccination or infection to sample collection (< 3, 3–6 vs. ≥ 6 months). To adjust for multiple testing for each analysis, all P-values were statistically significant with a Bonferroni correction.

Multivariate analyses were conducted to assess factors associated with humoral and cellular positive immune response. Age at sample collection (< 55 vs. ≥ 55 years), sex (male vs. female), disease type (myeloid malignancies vs. others), graft source (bone marrow vs. cord blood), chronic GVHD (none, non-active vs. active), IST (none vs. administration), interval from HCT to SARS-CoV-2 vaccination or infection (< 2 vs. ≥ 2 years), SARS-CoV-2 status (infection vs. vaccination), and interval from SARS-CoV-2 vaccination or infection to sample collection (< 6 vs. ≥ 6 months) were included as factors and evaluated using a logistic regression model.

## Results

### Patient characteristics

The clinical characteristics of the patients are shown in Table 1. Overall, 58 patients were included in this cross-sectional analysis. Their median age was 45.5 years (range, 10–68 years) at the time of HCT and 54.5 years (range, 19–72 years) at sample collection. Thirty-five patients (60%) were male. The most common disease type was acute myeloid leukemia (52%), and the most common graft type was unrelated CB (81%). Myeloablative conditioning regimens (96%) and cyclosporine and methotrexate for GVHD prophylaxis (66%) were commonly performed.

**Table 1:** Patient characteristics

Characteristic	Value
Number of patients	58
Median age at HCT, years (range)	45.5 (10–68)
Median age at sample collection, years (range)	54.5 (19–72)
Sex	
Male	35 (60%)
Female	23 (40%)
Disease type	
AML	30 (52%)
ALL	15 (26%)
MDS	8 (14%)
CMML	2 (3%)
ATLL	1 (2%)
MM	1 (2%)
SAA	1 (2%)
Donor/graft type	
Related bone marrow	7 (12%)
Unelated bone marrow	4 (7%)
Unrelated cord blood	47 (81%)
Conditioning regimen	
MAC	56 (96%)
RIC	1 (2%)
Unknown	1 (2%)
GVHD prophylaxis	
CSP + MTX	38 (66%)
CSP + MMF	12 (21%)
CSP	3 (5%)
TAC + MTX	3 (5%)
TAC + MMF	1 (2%)
Unknown	1 (2%)
Median period from HCT to sample collection, months (range)	75 (3–296)
Chronic GVHD at sample collection	
None/non-active chronic GVHD	18 (31%)
Active chronic GVHD	40 (69%)
Systemic immunosuppressive treatments at sample collection	
None	51 (88%)
Prednisolone and/or calcineurin inhibitors	7 (12%)

*HCT* hematopoietic cell transplantation, *AML* acute myelogenous leukemia, *ALL* acute lymphoblastic leukemia, *MDS* myelodysplastic syndrome, *CMML* chronic myelomonocytic leukemia, *ATLL* adult T-cell leukemia/lymphoma, *MM* multiple myeloma, *SAA* severe aplastic anemia, *MAC* myeloablative conditioning, *RIC* reduced-intensity conditioning, *GVHD* graft-versus-host disease, *CSP* cyclosporine A, *MTX* methotrexate, *MMF* mycophenolate mofetil, *TAC* tacrolimus

The median period from HCT to sample collection was 75 months (range, 3–296 months). At the time of sample collection, 40 patients (69%) had active chronic GVHD, and 7 patients (12%) had received IST for prophylaxis or treatment of chronic GVHD.

Before sample collection, 53 patients received two, three, or four doses of mRNA SARS-CoV-2 vaccine with either mRNA-1273 (Moderna) or BNT162b2 (Pfizer-BioNTech). Four patients had experienced polymerase chain reaction (PCR)-confirmed infection with SARS-CoV-2 after vaccination. One patient with no history of COVID-19 vaccination or infection developed cellular and humoral responses for SARS-CoV-2 after close contact with confirmed COVID-19 individuals, suggesting asymptomatic infection of COVID-19. The median period from HCT to SARS-CoV-2 vaccination or infection was 73.5 months (range, 4–293 months). The median period from SARS-CoV-2 vaccination or infection to sample collection was 110.5 days (range, 6–345 days).

### Humoral immunogenicity

**Fig. 1:**
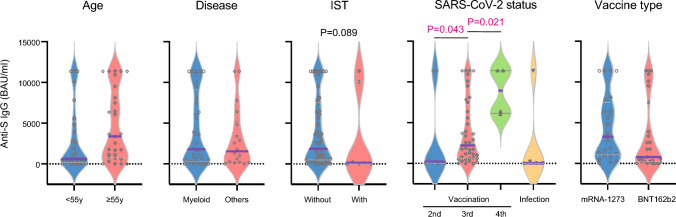
Anti-spike antibody level against SARS-CoV-2 in allogeneic HCT recipients according to the clinical characteristics of allogeneic HCT and SARS-CoV-2. *P* < 0.005 (0.05/10) was considered to be statistically significant with a Bonferroni correction. *P* values between 0.005 and 0.05 were considered to have a marginal significance

Among the whole cohort, the median antibody level was 1761 BAU/ml (range, 0 to > 11,360 BAU/ml). The antibody response was positive in 77.5% (45/58) of patients. There were no significant differences in antibody level by age (*P* = 0.105), gender (*P* = 0.342), disease type (*P* = 0.509), graft source (*P* = 0.814), chronic GVHD (*P* = 0.821), interval from HCT to SARS-CoV-2 vaccination or infection (*P* = 0.391), or vaccine type (*P* = 0.115). The antibody level after the third vaccination was marginally higher than after the second vaccination (*P* = 0.043). The antibody level after the fourth vaccination was marginally higher than after the third vaccination (*P* = 0.021). The antibody level was lower in patients who received IST compared to those who did not (*P* = 0.089), but this was not significant. The antibody level gradually decreased following SARS-CoV-2 vaccination or infection, but this was not significant (3–6 months vs. ≥ 6 months, *P* = 0.101) (Fig. 1, Supplementary Fig. 1a). Multivariate analysis showed that antibody positive response was associated with the administration of IST (Odd ratio, 0.03; *P* = 0.036) and SARS-CoV-2 vaccination (Odd ratio, 68.70; *P* = 0.004) (Supplementary Table 2).

### Cellular immunogenicity

**Fig. 2:**
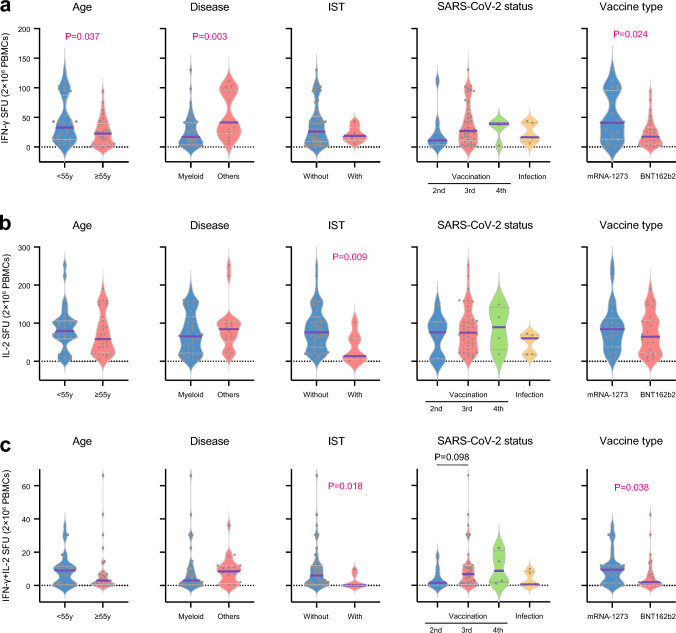
Frequencies of SARS-CoV-2 specific IFN-γ-producing T cells (**a**), IL-2-producing T cells (**b**), and IFN-γ and IL-2-producing T cells (**c**) in allogeneic HCT recipients according to the clinical characteristics of allogeneic HCT and SARS-CoV-2. *P* < 0.005 (0.05/10) was statistically significant with a Bonferroni correction. *P* values between 0.005 and 0.05 were considered to have a marginal significance

The median frequency of spike-specific IFN-γ-producing T cells was 25.2 SFU (range, 0.0–130.0 SFU). The median negative control and positive control by PMA/IM stimulation for IFN-γ-producing T cells was 4.1 SFU (range, 0.0–43.3 SFU) and 392.5 SFU (range, 23.0–670.6 SFU), respectively. The IFN-γ-producing T-cell response was positive in 68.9% (40/58) of patients. Older than 55 years of age (*P* = 0.037), myeloid disease (*P* = 0.003), and BNT162b2 (*P* = 0.024) were marginally associated with a lower frequency of spike-specific IFN-γ-producing T cells (Fig. 2a, Supplementary Fig. 1b). Multivariate analysis showed that IFN-γ-producing T-cell positive response was associated with females (Odd ratio, 4.79; *P* = 0.030) (Supplementary Table 2).

The median frequency of spike-specific IL-2-producing T cells was 70.2 SFU (range, 2.0–252.5 SFU). The median negative control and positive control by PMA/IM stimulation for IFN-γ-producing T cells was 16.8 SFU (range, 0.0–106.0 SFU) and 345.0 SFU (range, 136.0–744.0 SFU), respectively. The IL-2-producing T-cell response was positive in 62.0% (36/58) of patients. The median frequency of spike-specific IL-2-producing T cells was marginally lower in patients who received IST compared to those who did not (*P* = 0.009) (Fig. 2b, Supplementary Fig. 1c).

The median frequency of spike-specific IFN-γ + IL-2-producing T cells was 5.5 SFU (range, 0.0–66.0 SFU). The median negative control and positive control by PMA/IM stimulation for IFN-γ + IL-2-producing T cells was 0.0 SFU (range, 0.0–1.6 SFU) and 58.3 SFU (range, 1.0–141.0 SFU), respectively. The IFN-γ + IL-2-producing T-cell response was positive in 56.8% (33/58) of patients. Administration of IST (*P* = 0.018) and BNT162b2 (*P* = 0.038) was marginally associated with a lower frequency of spike-specific IFN-γ + IL-2-producing T cells (Fig. 2c, Supplementary Fig. 1d).

### Correlation of SARS-CoV-2 antibody levels and frequencies of SARS-CoV-2-specific T cells

**Fig. 3:**
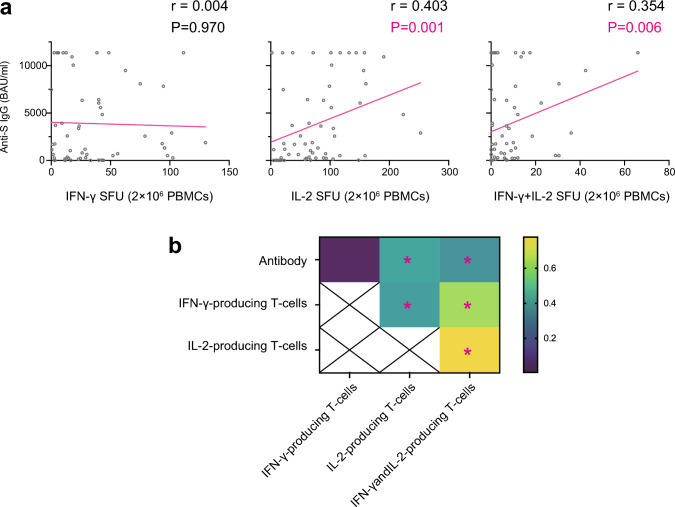
Correlations between SARS-CoV-2 specific antibody levels, IFN-γ-producing T cells, IL-2-producing T cells, and IFN-γ and IL-2-producing T cells in allogeneic HCT recipients (**a**). Heatmap of the correlations between cellular and humoral responses against SARS-CoV-2 (**b**). The color of the heatmap denotes the value of *r* as indicated in the color key. *P* < 0.008 (0.05/6) by the Spearman rank correlation coefficient was statistically significant with a Bonferroni correction. *P* values between 0.008 and 0.05 were considered to have a marginal significance. *P < 0.008

Correlation analysis was performed between SARS-CoV-2 antibodies and SARS-CoV-2-specific T cells. The antibody level was significantly correlated with IL-2-producing T cells (*P* = 0.001) and IFN-γ + IL-2-producing T cells (*P* = 0.006) but not IFN-γ-producing T cells (*P* = 0.970). However, the correlation between antibody level and IL-2-producing T cells (*r* = 0.403) and IFN-γ + IL-2-producing T cells (*r* = 0.354) was weak. The IFN-γ-producing T cells were also significantly correlated with IL-2-producing T cells (*r* = 0.384, *P* = 0.002) and IFN-γ + IL-2-producing T cells (*r* = 0.632, *P* < 0.0001). The IL-2-producing T cells were strongly and significantly correlated with IFN-γ + IL-2-producing T cells (*r* = 0.778, *P* < 0.0001) (Fig. 3).

### Correlation between peripheral blood lymphocyte subpopulations and SARS-CoV-2 antibody levels and frequencies of SARS-CoV-2-specific T cells

**Fig. 4:**
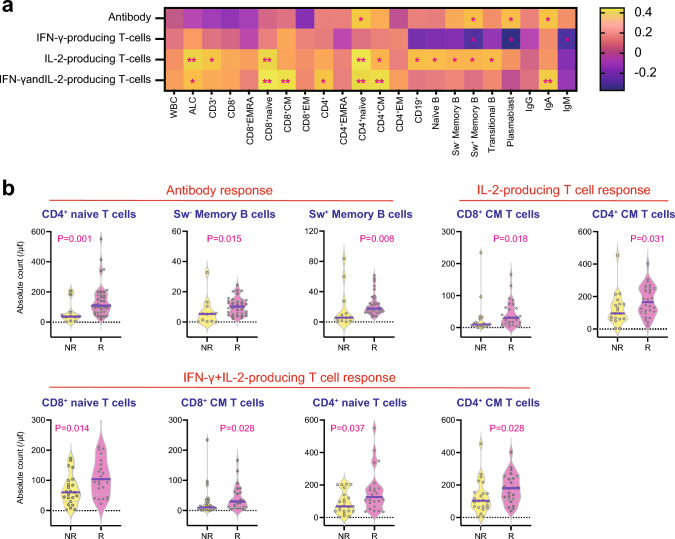
Heatmap of the correlations between cellular and humoral responses against SARS-CoV-2 and lymphocyte subpopulations in allogeneic HCT recipients (**a**). Absolute numbers of lymphocyte subpopulations according to the cellular and humoral responses against SARS-CoV-2 (**b**). **a** The color of the heatmap denotes the value of r as indicated in the color key. *P* < 0.002 (0.05/22) by the Spearman rank correlation coefficient was statistically significant with a Bonferroni correction. *P* values between 0.002 and 0.05 were considered to have a marginal significance. *P = 0.05 to 0.002, **P < 0.002. **b** *P* < 0.005 (0.05/9) was statistically significant with a Bonferroni correction. *P* values between 0.005 and 0.05 were considered to have a marginal significance. WBC, white blood cell; ALC, absolute lymphocyte count; EMRA, effector memory-expressing CD45RA; CM, central memory; EM, effector memory; Sw, switched; NR, non-responder; R, responder

We examined the simultaneous correlation between peripheral blood lymphocyte subpopulations and SARS-CoV-2 antibody levels and frequencies of SARS-CoV-2-specific T cells in 47 patients. The antibody level was positively associated with the absolute counts of CD4+ naïve T cells, switched memory B cells, and plasmablasts. In contrast, we found an inverse correlation between IFN-γ-producing T cells and the absolute counts of switched memory B cells and plasmablasts. IL-2- and IFN-γ + IL-2-producing T cells and lymphocyte subpopulations were also highly correlated: absolute lymphocyte counts, absolute counts of CD8+ naïve T cells, CD4+ naïve T cells, and CD4+ central memory T cells were positively correlated with both IL-2- and IFN-γ + IL-2-producing T cells. We also analyzed the correlation between serum immunoglobulin levels and SARS-CoV-2 antibody levels and frequencies of SARS-CoV-2-specific T cells. Serum IgA levels were positively associated with antibody levels and frequencies of IFN-γ + IL-2-producing T cells, but IgM levels were negatively associated with IFN-γ-producing T cells (Fig. 4a).

**Fig. 5:**
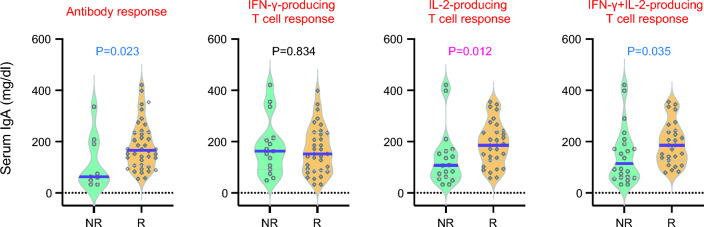
Serum IgA levels according to the cellular and humoral responses against SARS-CoV-2. *P* < 0.012 (0.05/4) was considered to be statistically significant with a Bonferroni correction. *P* values between 0.012 and 0.05 were considered to have a marginal significance. NR, non-responder; R, responder

When responders and non-responders were compared by the numbers of lymphocyte subpopulations, the absolute counts of CD4+ naïve T cells, non-switched memory B cells, and switched memory B cells were significantly or marginally higher in antibody responders compared with non-responders. There were no significant differences in all types of lymphocyte subpopulations between IFN-γ-producing cellular responders and non-responders. The absolute counts of CD8+ central memory T cells and CD4+ central memory T cells were marginally higher in IL-2-producing cellular responders compared with non-responders. Reflecting this, the absolute counts of CD8+ naïve T cells, CD8+ central memory T cells, CD4+ naïve T cells, and CD4+ central memory T cells were marginally higher in IFN-γ + IL-2-producing cellular responders compared with antibody non-responders (Fig. 4b). Serum IgA levels were significantly or marginally higher in antibody, IL-2-producing cellular, and IFN-γ + IL-2-producing cellular responders but not IFN-γ-producing cellular responders (Fig. 5).

### Comparison between the healthy control group and allogeneic HCT recipients

Finally, we evaluated the cellular and humoral immunogenicity against SARS-CoV-2 vaccination or infection and examine the correlation between lymphocyte subpopulations in peripheral blood and the cellular and humoral immunogenicity in 7 healthy subjects. The median age at sample collection was 48 years (range, 40–63 years), and two subjects were female. Among them, six subjects received four to seven doses of mRNA SARS-CoV-2 vaccine. One subject had experienced PCR-confirmed infection with SARS-CoV-2 after third vaccination. There were no significant differences in the antibody level, and frequency of spike-specific IFN-γ-producing T cells, spike-specific IL-2-producing T cells, and spike-specific IFN-γ + IL-2-producing T cells between the healthy control group and HCT recipients (Supplementary Fig. 2). Like allogeneic HCT recipients, there was a significant correlation between IL-2-producing T cells and IFN-γ + IL-2-producing T cells (*r* = 0.950, *P* = 0.001). The IFN-γ-producing T cells were also marginally correlated with IL-2-producing T cells (*r* = 0.839, *P* = 0.018) and IFN-γ + IL-2-producing T cells (*r* = 0.761, *P* = 0.046) (Supplementary Fig. 3a). Furthermore, among the healthy control group in the lymphocyte subpopulation analysis, there was a significant correlation between IL-2-producing T cells and the absolute counts of unswitched and switched memory B cells. Absolute numbers of CD4+ central and effector memory T cells and switched memory B cells were significantly correlated with IFN-γ + IL-2-producing T cells (Supplementary Fig. 3b).

## Discussion

We evaluated the cellular and humoral immunogenicity against SARS-CoV-2 vaccination or infection and the lymphocyte subpopulations in adults after allogeneic HCT. SARS-CoV-2 spike-specific antibody titer was significantly correlated with the frequencies of IL-2-producing T cells and IFN-γ + IL-2-producing T cells but not IFN-γ-producing T cells. In the lymphocyte subpopulation analysis, cellular and humoral immunogenicity against SARS-CoV-2 vaccination or infection was associated with the memory phenotype of B cells and T cells in this population.

Numerous studies have evaluated the longevity of cellular and humoral immunity against SARS-CoV-2 vaccination or infection. Antibody levels against SARS-CoV-2 decline rapidly over the first 4–6 months after vaccination or infection in the general population [51–55] in the same way that antibody levels to SARS-CoV-2 vaccination decline over time in allogeneic HCT recipients [56, 57]. In contrast, the cellular response against SARS-CoV-2 tends to remain stable in the general population [44, 45], but data on the longevity of cellular response are still scarce in adults receiving allogeneic HCT. Our cross-sectional study clearly demonstrated that SARS-CoV-2 specific IL-2-producing T cells are maintained up to 12 months after vaccination or infection, although antibody levels and IFN-γ-producing T cells decline over time in allogeneic HCT recipients. This is consistent with previous studies showing SARS-CoV-2-specific T-cell memory along with a predominant IL-2 memory response compared to IFN-γ in the general population [44, 45]. Furthermore, long-term SARS‐CoV‐2‐specific memory T-cell responses generate both IFN-γ and IL-2 following mild to severe COVID‐19 [58]. Collectively, these data suggest that SARS-CoV-2-specific IL-2-and IFN-γ + IL-2-producing T cells play a fundamental role in the longevity of cellular and humoral immunity against SARS-CoV-2 vaccination or infection not only in the general population but also in allogeneic HCT recipients.

Among allogeneic HCT recipients, we found that humoral responders were characterized by an increased frequency of CD4+ naïve T cells and switched memory B cells compared with non-responders. Several studies have shown these correlations as follows [59–61]: for example, a larger CD4+ naïve T-cell pool causes the SARS-CoV-2 specific T-cell response to happen more quickly [59] and induces a more robust SARS-CoV-2 specific antibody response. Indeed, the severity of COVID-19 is correlated with a lower frequency of naive T cells [59, 60]. Finally, switched memory B cells quickly develop into antibody-secreting cells after a series of viral vaccinations or infections [61]. In regard to the T-cell response, our data showed that the absolute counts of CD8+ and CD4+ central memory T cells were higher in both IL-2- and IFN-γ + IL-2-producing cellular responders compared with non-responders. Our findings suggest that SARS‐CoV‐2 specific memory T cells may have a central memory T phenotype that produces IL-2 [61, 62]. Furthermore, there are SARS-CoV-2 cross-reactive and IL-2 secreted T cells in contacts of confirmed COVID-19 cases [63], which suggests that higher central memory T-cell counts might be positively associated with pre-existing SARS-CoV-2 cross-reactive T cells. However, these studies have also shown these correlations in the general population [59–63]. In line with allogeneic HCT recipients, our data also showed that both IL-2- and IFN-γ + IL-2-producing cellular responses against SARS-CoV-2 vaccination or infection were associated with the memory phenotype of T- and B-lymphocytes among healthy subjects without hematological disease. These observations suggest the similarities between immunogenicity against SARS-CoV-2 and lymphoid subpopulations among the general population and allogeneic HCT recipients.

Our study also showed that serum total IgA levels were significantly higher in SARS-CoV-2 specific humoral, IL-2-producing cellular, and IFN-γ + IL-2-producing cellular responders. Mucosal immunity has been shown to be important for protection against SARS-CoV-2 infection [64, 65]. Although our data could not confirm the correlation between serum total IgA levels and SARS-CoV-2 specific secretary IgA levels in saliva, previous studies found that SARS-CoV-2 specific IgA levels in the serum were positively correlated with matched saliva samples in the general population [66]. Furthermore, secretory IgA, particularly dimers, has a strong potential for SARS-CoV-2 neutralization [67]. These findings suggest that SARS-CoV-2 specific adaptive cellular and humoral responses might be reflected in secretary IgA levels in saliva. Further studies are needed to clarify the role of mucosal immunity against SARS-CoV-2 in immunocompromised patients, particularly allogeneic HCT recipients.

Our study had several limitations. First, its cross-sectional design limited the comparison of SARS-CoV-2 vaccine effectiveness across the studies, because the number of vaccinations and the duration from vaccination to evaluation varied among individuals in our study, which could have affected the SARS-CoV-2 specific antibody level and the frequency of T cells. Moreover, the median period from HCT to sample collection was 75 months, indicating that it seems to be too long to considered patients as a representative of short-term HCT recipients, but it included long-term survivors with a median follow-up period of 6 years. Second, different positivity cutoff values of antibody titer and the frequency of T cells by ELISpot assay were used across the studies and assays used. Regardless of the cutoff level and method of evaluation of cellular and humoral immunogenicity, the patients who received allogeneic HCT with long-term follow-up may well acquire both cellular and humoral immunity to SARS-CoV-2, one of this study’s main findings. Third, we could not assess the lymphocyte subpopulations of cytokine-producing T cells specific to SARS-CoV-2 in this study. Therefore, further studies are needed to clarify lymphocyte subpopulations against SARS-CoV-2 vaccination or infection. Fourth, a recent study from Russia demonstrated that the contribution of SARS-CoV-2-specific antibodies is more pronounced for protection against SARS-CoV-2 infection than that of T cells in the general population [68], suggesting that quantitative measurement of antibody concentration in serum is advantageous for the prediction of protection against SARS-CoV-2 infection. However, the levels of SARS-CoV-2-specific antibodies and T cells required to protect allogeneic HCT recipients from SARS-CoV-2 infection are unclear. Fifth, in Japan, this study was performed during the Omicron epidemic waves, including BA.1, BA2, and BA4/5. Unfortunately, the immunogenicity against new variants of concern (VoCs) that mainly accumulate mutations in the spike protein could not be evaluated in our study. However, recent studies demonstrated that SARS-CoV-2 vaccine-induced T cells have cross-reactivity against VoCs despite the evasion of neutralizing antibodies [69, 70].

In summary, the patients who received allogeneic HCT may well acquire both cellular and humoral immunity to SARS-CoV-2 vaccination or infection. This cellular and humoral immunogenicity against SARS-CoV-2 vaccination or infection was associated with the memory type of T cells and B cells in allogeneic HCT recipients.

### Supplementary Information

Below is the link to the electronic supplementary material.

Supplementary file1 (DOCX 14 KB)

Supplementary file2 (DOCX 21 KB)

Supplementary file3 (PDF 3606 KB)

Supplementary file4 (PDF 1011 KB)

Supplementary file5 (PDF 480 KB)

## Data Availability

Data may be available from the corresponding author upon reasonable request.
