# Supplementary material for: Cellular and humoral immunogenicity against SARS-CoV-2 vaccination or infection is associated with the memory phenotype of T- and B-lymphocytes in adult allogeneic hematopoietic cell transplant recipients
Source: Int J Hematol. 2024 Jun 6;120(2):229–40. doi: 10.1007/s12185-024-03802-3 (PMC11284193; doi:10.1007/s12185-024-03802-3)
Supplement: Supplementary file 1 — Supplementary file1 (DOCX 14 KB) [file 12185_2024_3802_MOESM1_ESM.docx]

**Supplementary Table 1. Surface markers to define lymphocyte subpopulations.**

| Lymphocyte subpopulations | Surface markers |
| --- | --- |
| T cell | CD3+ |
| CD8+ | CD3+ CD8+ |
| CD8+EMRA | CD3+ CD8+CCR7-CD45RA+ |
| CD8+naïve | CD3+ CD8+CCR7+CD45RA+ |
| CD8+CM | CD3+ CD8+CCR7+CD45RA- |
| CD8+EM | CD3+ CD8+CCR7-CD45RA- |
| CD4+ | CD3+ CD4+ |
| CD4+EMRA | CD3+ CD4+CCR7-CD45RA+ |
| CD4+naïve | CD3+ CD4+CCR7+CD45RA+ |
| CD4+CM | CD3+ CD4+CCR7+CD45RA- |
| CD4+EM | CD3+ CD4+CCR7-CD45RA- |
| B cell | CD3-CD19+ |
| Naïve B | CD3-CD19+IgD+CD27- |
| Non-switched memory B | CD3-CD19+IgD+CD27+ |
| Switched memory B | CD3-CD19+IgD-CD27+ |
| Transitional B | CD3-CD19+CD24+CD38+ |
| Plasmablast | CD3-CD19+CD27+CD38+ |

EMRA, effector memory-expressing CD45RA; CM, central memory; EM, effector memory.
