# Supplementary material for: Cellular and humoral immunogenicity against SARS-CoV-2 vaccination or infection is associated with the memory phenotype of T- and B-lymphocytes in adult allogeneic hematopoietic cell transplant recipients
Source: Int J Hematol. 2024 Jun 6;120(2):229–40. doi: 10.1007/s12185-024-03802-3 (PMC11284193; doi:10.1007/s12185-024-03802-3)
Supplement: Supplementary file 2 — Supplementary file2 (DOCX 21 KB) [file 12185_2024_3802_MOESM2_ESM.docx]

**Supplementary Table 2.** Multivariate analysis of factors associated with humoral and cellular positive immune response.

|  | Antibody response |  | IFN-γ-producing T cell response |  | IL-2-producing T cell response |  | IFN-γ+ IL-2-producing T cell response |  |
| --- | --- | --- | --- | --- | --- | --- | --- | --- |
|  | Odd ratio (95% CI) | P value | Odd ratio (95% CI) | P value | Odd ratio (95% CI) | P value | Odd ratio (95% CI) | P value |
| Age |  |  |  |  |  |  |  |  |
| < 55 years | Reference |  | Reference |  | Reference |  | Reference |  |
| ≥ 55 years | 0.49 (0.04-5.11) | 0.555 | 0.33 (0.07-1.53) | 0.159 | 0.23 (0.05-1.10) | 0.065 | 0.41 (0.09-640.00) | 0.874 |
| Sex |  |  |  |  |  |  |  |  |
| Male | Reference |  | Reference |  | Reference |  | Reference |  |
| Female | 5.56 (0.48-64.00) | 0.169 | 4.79 (1.16-19.70) | **0.030** | 1.19 (0.33-4.27) | 0.789 | 0.49 (0.14-1.71) | 0.268 |
| Disease type |  |  |  |  |  |  |  |  |
| Myeloid | Reference |  | Reference |  | Reference |  | Reference |  |
| Others | 0.32 (0.04-2.10) | 0.235 | 0.83 (0.20-3.49) | 0.806 | 1.03 (0.24-4.32) | 0.967 | 1.57 (0.38-6.36) | 0.530 |
| Graft source |  |  |  |  |  |  |  |  |
| BM | Reference |  | Reference |  | Reference |  | Reference |  |
| CB | 1.14 (0.03-38.40) | 0.940 | 1.65 (0.26-10.30) | 0.589 | 0.39 (0.05-2.99) | 0.367 | 0.90 (0.15-5.28) | 0.914 |
| cGVHD |  |  |  |  |  |  |  |  |
| None | Reference |  | Reference |  | Reference |  | Reference |  |
| Active | 2.62 (0.29-23.80) | 0.391 | 1.27 (0.29-5.41) | 0.745 | 0.47 (0.11-1.95) | 0.301 | 0.34 (0.08-1.38) | 0.132 |
| IST |  |  |  |  |  |  |  |  |
| None | Reference |  | Reference |  | Reference |  | Reference |  |
| Administration | 0.03 (0.001-0.80) | **0.036** | 2.89 (0.19-44.00) | 0.445 | 0.40 (0.03-4.87) | 0.475 | 0.17 (0.01-1.81) | 0.145 |
| Interval from HCT to vaccination/infection |  |  |  |  |  |  |  |  |
| < 2 years | Reference |  | Reference |  | Reference |  | Reference |  |
| ≥ 2 years | 4.51 (0.60-33.60) | 0.142 | 0.89 (0.19-4.13) | 0.881 | 0.95 (0.22-3.99) | 0.946 | 1.43 (0.35-5.82) | 0.616 |
| SARS-CoV-2 status |  |  |  |  |  |  |  |  |
| Infection | Reference |  | Reference |  | Reference |  | Reference |  |
| Vaccination | 68.70 (3.64-1300.00) | **0.004** | 2.12 (0.25-18.00) | 0.490 | 1.46 (01.7-12.60) | 0.729 | 2.30 (0.29-18.00) | 0.428 |
| Interval from vaccination/infection to evaluation |  |  |  |  |  |  |  |  |
| < 6 months | Reference |  | Reference |  | Reference |  | Reference |  |
| ≥ 6 months | 0.47 (0.025-9.07) | 0.621 | 0.64 (0.05-6.89) | 0.714 | 0.51 (0.05-4.86) | 0.563 | 0.73 (0.07-7.09) | 0.791 |

BM, bone marrow; CB, cord blood; cGVHD, chronic graft-versus-host disease; IST, immunosuppressive treatment; HCT, hematopoietic cell transplantation.

The P values in red bold are statistically significant (<.05).
