# Supplementary material for: Cellular and humoral immunogenicity against SARS-CoV-2 vaccination or infection is associated with the memory phenotype of T- and B-lymphocytes in adult allogeneic hematopoietic cell transplant recipients
Source: Int J Hematol. 2024 Jun 6;120(2):229–40. doi: 10.1007/s12185-024-03802-3 (PMC11284193; doi:10.1007/s12185-024-03802-3)
Supplement: Supplementary file 4 — Supplementary file4 (PDF 1011 KB) [file 12185_2024_3802_MOESM4_ESM.pdf]

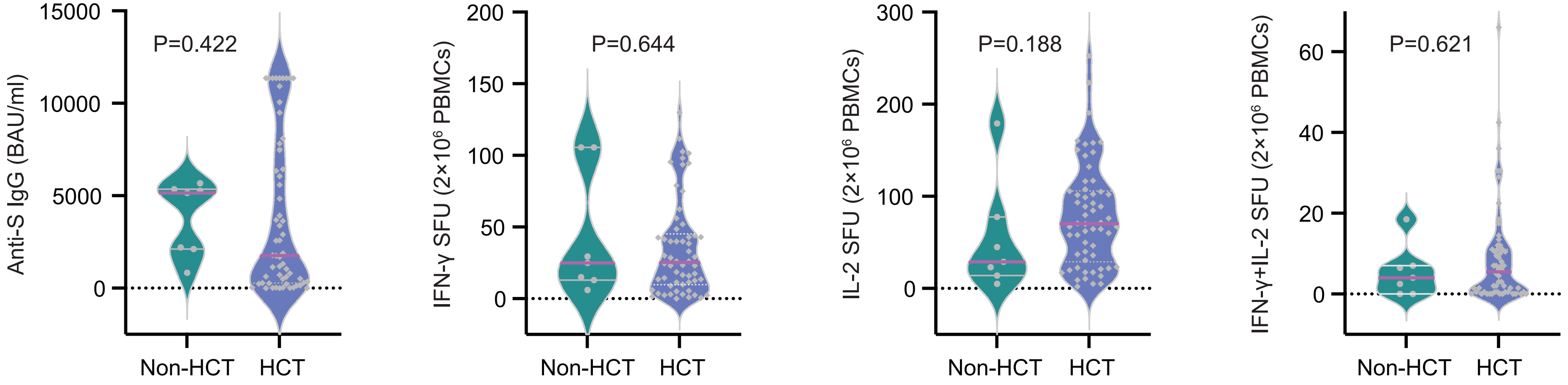

Supplementary Figure 2. Anti-spike antibody level against SARS-CoV-2, and frequencies of SARS-CoV-2 specific IFN-γ-producing T cells, IL-2-producing T cells, and IFN-γ and IL-2-producing T cells in healthy control (non-HCT, n=7) and allogeneic HCT recipients (n=58).
