# Supplementary material for: Cellular and humoral immunogenicity against SARS-CoV-2 vaccination or infection is associated with the memory phenotype of T- and B-lymphocytes in adult allogeneic hematopoietic cell transplant recipients
Source: Int J Hematol. 2024 Jun 6;120(2):229–40. doi: 10.1007/s12185-024-03802-3 (PMC11284193; doi:10.1007/s12185-024-03802-3)
Supplement: Supplementary file 5 — Supplementary file5 (PDF 480 KB) [file 12185_2024_3802_MOESM5_ESM.pdf]

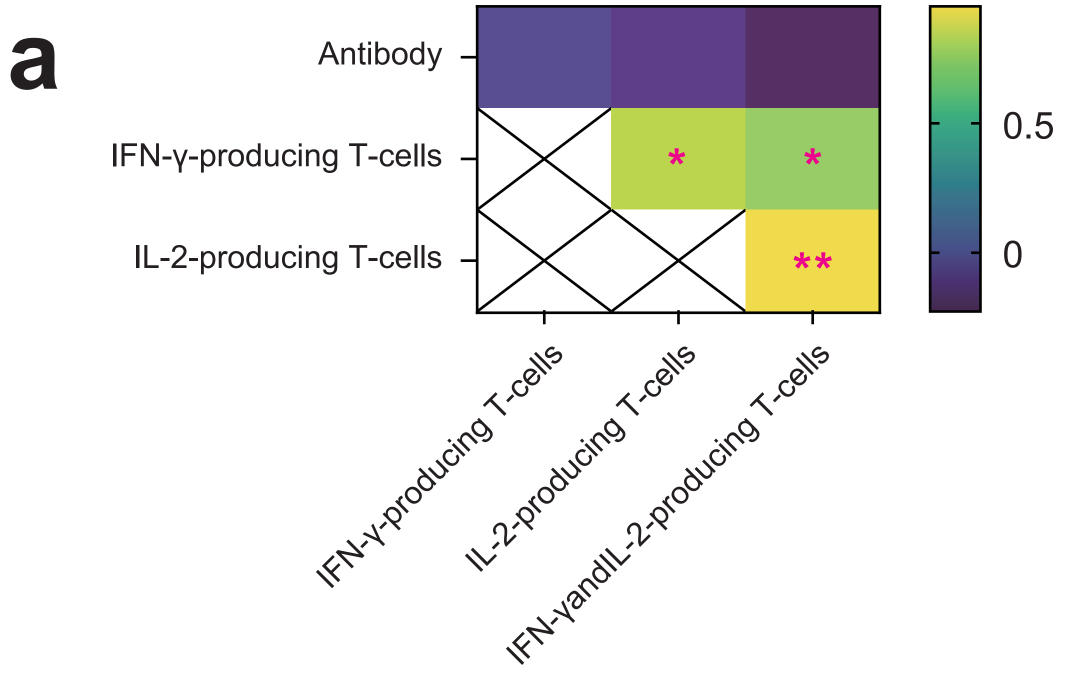

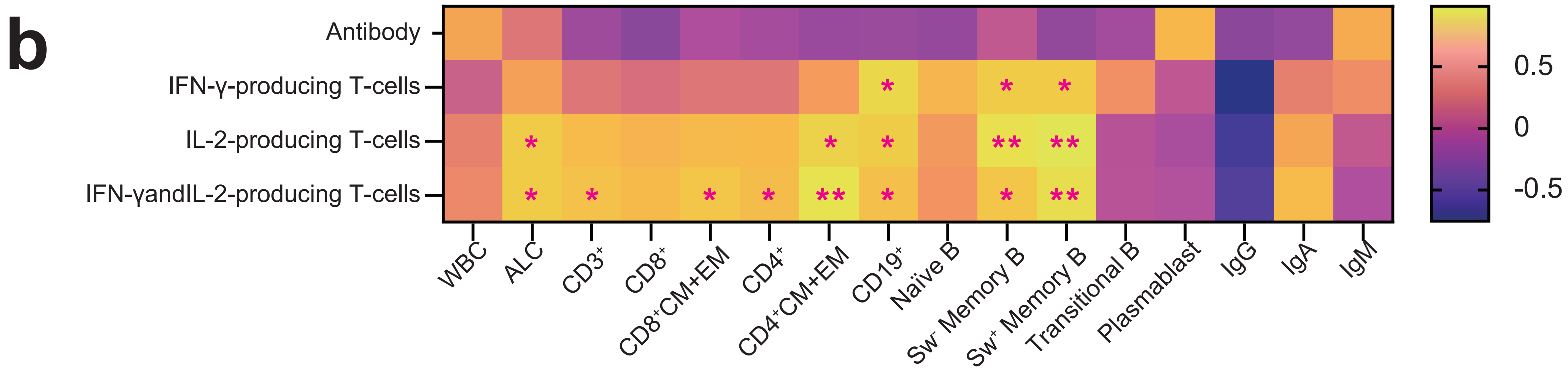

Supplementary Figure 3. Heatmap of the correlations between cellular and humoral responses against SARS-CoV-2 (a), and the correlations between cellular and humoral responses against SARS-CoV-2 and lymphocyte subpopulations (b) in healthy control.

The color of the heatmap denotes the value of r as indicated in the color key.

(a) P < 0.008 (0.05/6) by the Spearman rank correlation coefficient was statistically significant with a Bonferroni correction. P values between 0.008 and 0.05 were considered to have a marginal significance. *P = 0.05 to 0.008, **P < 0.008. (b) P < 0.003 (0.05/16) by the Spearman rank correlation coefficient was statistically significant with a Bonferroni correction. P values between 0.003 and 0.05 were considered to have a marginal significance.

*P = 0.05 to 0.003, **P < 0.002.
